# Feasibility and Diagnostic Accuracy of Ischemic Stroke Territory Recognition Based on Two-Dimensional Projections of Three-Dimensional Diffusion MRI Data

**DOI:** 10.3389/fneur.2015.00239

**Published:** 2015-11-19

**Authors:** Jana Katharina Wrosch, Bastian Volbers, Philipp Gölitz, Daniel Frederic Gilbert, Stefan Schwab, Arnd Dörfler, Johannes Kornhuber, Teja Wolfgang Groemer

**Affiliations:** ^1^Department of Psychiatry and Psychotherapy, Friedrich-Alexander University of Erlangen-Nuremberg, Erlangen, Germany; ^2^Department of Neurology, Friedrich-Alexander University of Erlangen-Nuremberg, Erlangen, Germany; ^3^Department of Neuroradiology, Friedrich-Alexander University of Erlangen-Nuremberg, Erlangen, Germany; ^4^Institute of Medical Biotechnology, Friedrich-Alexander University of Erlangen-Nuremberg, Erlangen, Germany; ^5^Psychiatric and Neurological Ambulatory Care Office, Bamberg, Germany

**Keywords:** computer-aided detection and diagnosis, diffusion-weighted imaging, dimensionality reduction, magnetic resonance imaging, stroke territories, validation, visualization

## Abstract

This study was conducted to assess the feasibility and diagnostic accuracy of brain artery territory recognition based on geoprojected two-dimensional maps of diffusion MRI data in stroke patients. In this retrospective study, multiplanar diffusion MRI data of ischemic stroke patients was used to create a two-dimensional map of the entire brain. To guarantee correct representation of the stroke, a computer-aided brain artery territory diagnosis was developed and tested for its diagnostic accuracy. The test recognized the stroke-affected brain artery territory based on the position of the stroke in the map. The performance of the test was evaluated by comparing it to the reference standard of each patient’s diagnosed stroke territory on record. This study was designed and conducted according to Standards for Reporting of Diagnostic Accuracy (STARD). The statistical analysis included diagnostic accuracy parameters, cross-validation, and Youden Index optimization. After cross-validation on a cohort of 91 patients, the sensitivity of this territory diagnosis was 81% with a specificity of 87%. With this, the projection of strokes onto a two-dimensional map is accurate for representing the affected stroke territory and can be used to provide a static and printable overview of the diffusion MRI data. The projected map is compatible with other two-dimensional data such as EEG and will serve as a useful visualization tool.

## Introduction

Worldwide, ischemic stroke is the third-most common cause of years of live lost (1). In developed countries, stroke even ranks second (1). Routine patients presenting with stroke symptoms are typically evaluated by assessing diffusion MRI data, which is the most accurate method of illustrating the affected brain area in the acute phase of a stroke (2). In diffusion MRI, the degree to which water molecules can diffuse freely in the tissue is measured. In brain regions affected by stroke, the diffusion of water is impaired and can be detected as a reduced darkening of the MRI image over a recording time of (typically) 20 ms per layer (3). To evaluate the results, the two-dimensional images are stacked to form a three-dimensional dataset. Through this method of representing the imaging data, an observer can only assess the full information through visual inspection and steady rotation or direct manipulation of the images (e.g., scrolling through the layers). This approach is not always possible and limits the applicability of the method in printed reports.

Computer-aided diagnosis of stroke has gained in importance over the last decade. There is a multitude of approaches (4–7). Most groups use CT images to make early developments of stroke visible and detectable (8–10). Tyan et al. (11) considered diffusion MRI data as well as CT images for unsupervised computer-aided diagnosis of stroke-affected brain volume. The focus of all these approaches lies in enhancing the imaging data so that search algorithms can detect stroke symptoms. With this work, we aim to add to the visualization possibilities, which are not addressed by most computer-aided diagnosis approaches. The dimensionality reduction to a two-dimensional depiction of the entire brain provides advantages in print, in compatibility with brain surface measurements such as EEG and in tracking changes of the stroke lesion over time. We here present a new approach of converting the three-dimensional imaging data set to a two-dimensional map by geoprojection. To ensure the correct representation of strokes in the two-dimensional map, we developed a computer-aided artery territory recognition algorithm, using the projected maps as input.

The projection of three-dimensional data into a two-dimensional map via Mollweide geoprojection is a common method in other fields (12–14). We applied this mathematical method to diffusion MRI data to create a static, two-dimensional representation of the entire brain providing accurate information on stroke position and size.

The purpose of this retrospective study was to develop an intuitive two-dimensional representation of three-dimensional brain imaging data and to probe it for its diagnostic validity. To validate the projection outcome, we assessed the diagnostic accuracy of computer-aided stroke territory recognition based on geoprojected two-dimensional maps of diffusion MRI data.

## Materials and Methods

### Study Population and Design

This retrospective study was designed and conducted according to Standards for Reporting of Diagnostic Accuracy (STARD) (15). The data for this study were collected using the institutional database of the Neurological Department at the Friedrich–Alexander University of Erlangen-Nuremberg. The patient’s demographic information was obtained from medical records. Patients of any sex and age were accepted if diffusion-weighted MR imaging data with apparent ischemic stroke was available. The patients’ stroke diagnoses on record were categorized according to the affected brain artery territory into the following groups: the anterior cerebral artery (ACA) territory, superior or inferior division of the middle cerebral artery (MCA) territory, the posterior cerebral artery (PCA) territory or the posterior inferior cerebellar artery (PICA) territory. This division of the brain follows the definition of vascular territories used in other studies (16–18). Superior cerebellar artery infarctions were rare in our search group; thus, this type of infarction was not included in the study.

### Test Methods

To report the diagnostic accuracy of the projected representation of stroke territories in two-dimensional maps, we used each of the diagnoses on record for the 125 patients in the study, which were approved by an experienced neurologist and neuroradiologist as a reference standard. The clinical diagnoses were based on the evaluation of the same diffusion MRI datasets used for our test method.

### Diffusion-Weighted MR Imaging

Image acquisition was performed on a 1.5-T MR-Scanner (Aera; Siemens, Erlangen, Germany) using routine protocols for stroke imaging, including an axial diffusion-weighted echo planar sequence (*b* value = 1000 s/mm^2^; repetition time = 6800 ms; echo time = 89 ms; slices = 25; slice thickness = 5 mm).

### Projection Method

The projection of MRI data into two-dimensional maps, as well as statistical analysis, was performed with MATLAB software (Mathworks). The scripts are available at https://github.com/janawrosch/mrt_projection.

To create the two-dimensional maps of the entire brain, we first arranged all diffusion MRI data points to form a three-dimensional data stack, which was similar to the stacked image layers commonly created for MRI evaluation.

**Figure 1:**
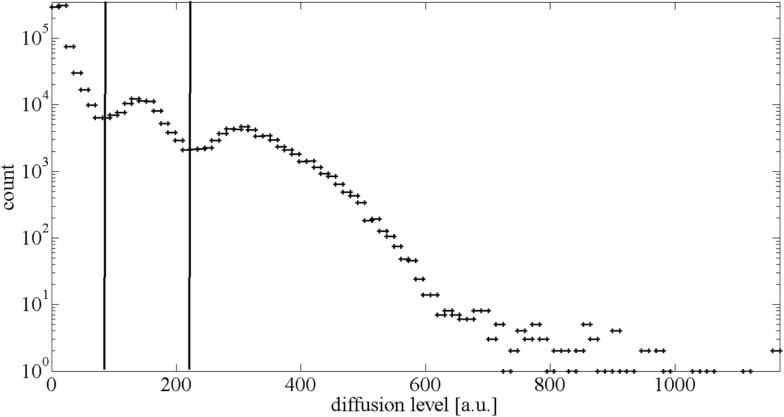
**Histogram of diffusion MRI values of one exemplary dataset**. The intense peak at the diffusion level around 20 a.u. represents the background. The broad peak around 180 a.u. representing the healthy brain values is accompanied by a smaller second “peak” around 300 a.u., which is representing the stroke-affected tissue. The left vertical line shows the calculated cut-off value between the background and the brain. The right vertical line shows the cut-off value between the healthy brain and stroke-affected tissue.

In the first step, the voxels depicting the brain were distinguished from the ones showing the background by background determination-based detection (19). For that, we plotted the values of all diffusion MRI data points from all layers into a histogram (Figure 1). This plot resulted in a large peak at low intensity values, which represents the background, and a smaller peak at higher intensities, which represents the brain. For the cut-off value between the background and brain, the minimum of the dip between those peaks was calculated (left line in Figure 1). This method is based on the assumption of a Gaussian distribution of the values in the histogram. Since it has been shown that MRI data follows a Rician distribution (20–22), we ensured applicability of the background determination-based method with the signal-to-noise levels at hand by testing the distributions in the histogram via Kolmogorov–Smirnov test for Gaussian distribution and found that it is applicable in each of the 125 cases (Figure S1 in Supplementary Material).

To evaluate the quality of the two-dimensional projection, the proportion of stroke volume per total brain volume in the diffusion MRI data was compared to the proportion of stroke area per total brain area in the two-dimensional map. For the separation of MRI voxels representing healthy brain from stroke voxels representing stroke-affected tissue, we also used the intensity histogram in which the broad peak representing the brain is positively skewed by the scarce high intensity stroke values. Based on a normal, and therefore symmetrical, distribution of healthy brain values, the cut-off point between the healthy brain and stroke is the minimum value from the left side of the curve reflected across the maximum of the healthy brain values, which is indicated by the right line in Figure 1. These cut-off values were calculated for each patient’s individual histogram.

**Figure 2:**
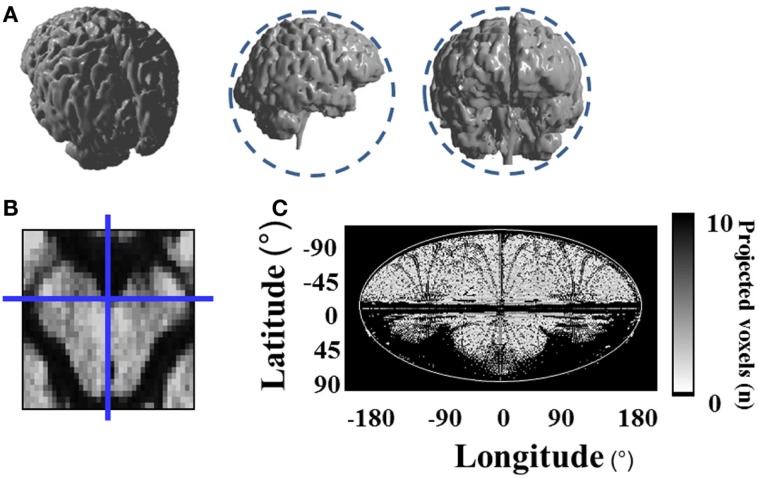
**Projection of three-dimensional diffusion MRI data onto a two-dimensional map**. **(A)** All data points are projected onto a sphere around the surface of the brain, which is illustrated by the dashed circles; **(B)** center of the projection sphere at the ventral surface of the lower mesencephalon between both cerebral peduncles; **(C)** plane transformation of the projection sphere via Mollweide geoprojection.

In the next step, all diffusion MRI data points representing the brain (healthy and stroke-affected tissue) were projected onto a sphere around the surface of the brain (see Figure 2A). For the center of this sphere, we chose the ventral surface of the lower mesencephalon between the cerebral peduncles, as shown in Figure 2B. We chose this location due to its centric and distinct position within the brain. The voxels within the sphere are projected straight out onto their nearest neighbor on the sphere. The generated surface contains all the information included in the three-dimensional MRI data volume. In a final step, this surface was transformed into a plane.

We used the mathematical method of Mollweide geoprojection (23) to convert the spherical coordinates λ and θ of each point on the sphere with radius r to Cartesian coordinates *x* and *y* using the following formulae:

$$x=\left(\sqrt{8}\cdot r\cdot \lambda \cdot \cos\theta \right)∕\pi ;\;y=\sqrt{2}\cdot r\cdot \sin\theta$$

With this geoprojection, the position of each data point on the sphere is transformed to a point on a two-dimensional map of the brain (see Figure 2C). In about 40% of the pixels on the map, it is the case that several data points fall into the range of the same pixel. In those pixels, the maximum diffusion value was plotted onto the map to ensure that no stroke data (higher values) gets lost or obstructed by healthy brain data (lower values).

**Figure 3:**
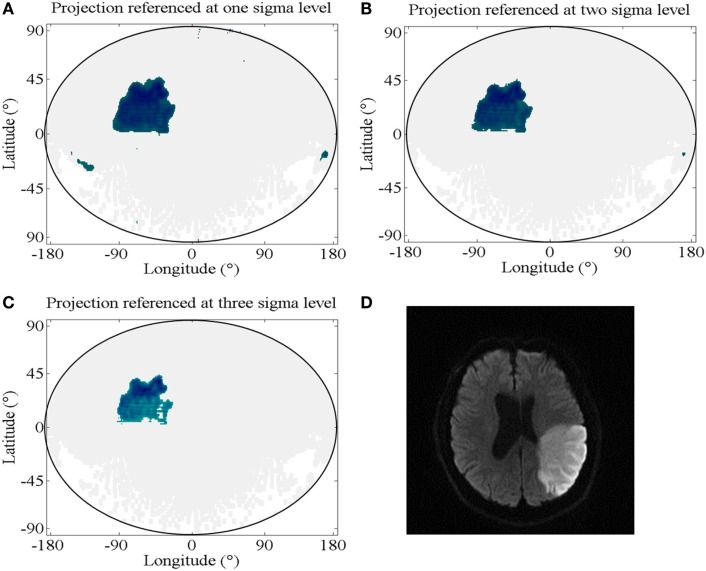
**Diffusion level reference**. **(A–C)** Projections with strokes indicated for the pixels of values above the mean plus different numbers of SDs from the healthy diffusion levels; **(A)** projection referenced at one sigma level, which corresponds to a mean difference in the proportion of stroke per total brain in the original MRI data and in the projected maps (SPB-ratio) of 16.5% (SD: 31.9%); **(B)** projection referenced at two sigma levels, which corresponds to a SPB-ratio of 5.4% (SD: 8.1%); **(C)** projection referenced at three sigma levels, which corresponds to a SPB-ratio of 9.2% (SD: 6.9%); **(D)** representative layer of the diffusion MRI data of the projected brain in **(A)** through **(C)**, showing the stroke-affected region.

To determine which diffusion levels indicate a stroke at each point on the map, we created a diffusion level reference map from the healthy brain hemispheres of 50 patients. For each single pixel of the map, we averaged the diffusion values of healthy brains and calculated the SD of the diffusion values in that point. A patient’s projected data are compared, pixel-wise, to this reference map. If the data differ by more than 2 SDs from the averaged healthy diffusion value at that point, the deviation is considered indicative of stroke. We determined the threshold of the mean plus 2 SDs by minimizing the difference in the proportion of the stroke-affected volume per total brain volume and the proportion of the stroke-affected area per total brain area in the two-dimensional projection, as shown in Figure 3.

With this software, the three-dimensionally stacked MRI data were automatically converted into a two-dimensional map showing the entire brain at once and indicating the stroke area with above threshold values.

**Figure 4:**
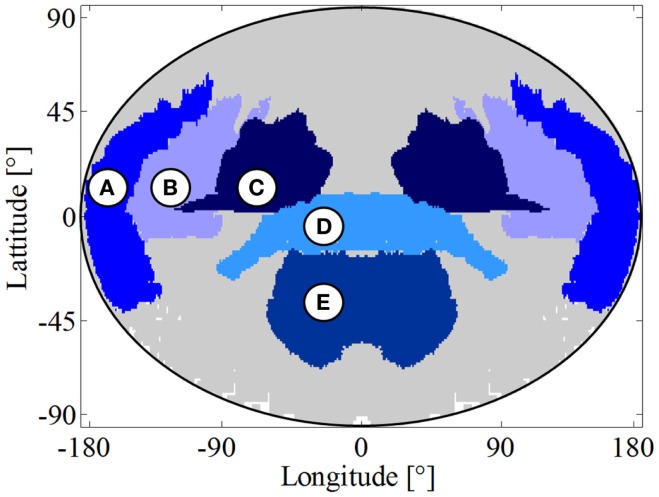
**Stroke territory reference map**. **(A)** Anterior cerebral artery territory; **(B)** superior division of the middle cerebral artery territory; **(C)** inferior division of the middle cerebral artery territory; **(D)** posterior cerebral artery territory; **(E)** posterior inferior cerebellar artery territory; left side of the map also shows the left side of the brain.

To be able to assign stroke coordinates in the two-dimensional map to a defined region in the patients’ brains, we created a stroke territory reference map referencing the pixels according to brain artery territory. We created this map by merging the stroke-covered regions of the patients with a distinct diagnosis of stroke in only the one artery territory. For good coverage of the complete artery territory, we chose patients who had a widespread lesion within that territory (see Figure 4). The patients used for this stroke territory assessment (14 patients) were excluded from further analysis to prevent biased test results.

To ensure accurate representation of strokes in the two-dimensional maps, we developed an algorithm for computer-aided stroke territory diagnosis that is using projected maps as input and assessed this test’s diagnostic accuracy.

The algorithm tests each territory for stroke by applying two criteria: (1) If a large percentage of the territories’ area is covered by stroke-indicating pixels, that territory is positive for stroke. The cut-off value for this criterion decides to what extend each region needs to be covered to be counted as positive. (2) If a large bulk of all stroke-indicating pixels falls together into the same territory, that territory is positive for stroke. The cut-off value for this criterion decides how many of all the stroke-indicating pixels need to be convened in a territory for this to be counted as positive. The values for each criterion and each round of outer cross-validation ore listed in Table 1.

**Table 1:** **Cut-off values for computer-aided brain artery territory diagnosis**.

Excluded groups	How much area must be covered by stroke?					How many of the stroke pixels must be convened?				
	ACA (%)	supMCA (%)	infMCA (%)	PCA (%)	PICA (%)	ACA (%)	supMCA (%)	infMCA (%)	PCA (%)	PICA (%)
1	20	10	5	10	30	0	0	0	90	50
2	20	10	5	10	30	0	0	0	90	50
3	20	10	5	10	30	0	0	0	90	50
4	20	10	5	10	30	0	0	0	90	50
5	20	10	5	10	30	0	0	0	90	50
6	20	10	5	10	30	0	0	0	90	50
7	20	10	5	10	25	0	0	0	90	50
8	20	10	5	10	30	0	0	0	90	50
9	20	10	5	10	30	0	0	0	25	50
10	20	10	5	10	30	0	0	0	90	50

*Best cut-off values were found by Youden Index maximization as shown in Figure S1 in Supplementary Material. The table gives the cut-off values for each criterion and each of the 10 rounds of leave-p-out cross-validation, where the optimization is based on all 10 subgroups of data except the named excluded group*.

*ACA, anterior cerebral artery territory; supMCA, superior division of the middle cerebral artery territory; infMCA, inferior division of the middle cerebral artery territory; PCA, posterior cerebral artery territory; PICA, posterior inferior cerebellar artery territory*.

The resulting stroke territory diagnosis was compared to the patient’s recorded clinical diagnosis (reference standard).

### Statistical Methods

To assess sensitivity and specificity (24) as well as the positive predictive value (PPV) and negative predictive value (NPV) and positive likelihood ratio (PLR) and negative likelihood ratio (NLR), we used leave-p-out cross-validation (25–27). The study population of 91 patients was randomly divided into 10 subgroups. In 10 rounds of parameter optimization and subsequent test validation one subgroup at a time was excluded from the parameter optimization. The following validation was applied to the excluded data set respectively. The test performance results of all 10 validation rounds were averaged. Parameter optimization as well as test performance evaluation was based on the counts of true positive (TP), false positive (FP), true negative (TN), and false negative (FN) results.

Test parameters were optimized to find the best cut-off values for the brain artery territory recognition criteria. Optimization was achieved by maximizing the Youden Index *Y* (28), which is calculated by the formula:

$$Y=\;\frac{\text{TP}}{\text{TP}+\text{FN}}+\frac{\text{TN}}{\text{FP}+\text{TN}}-1$$

The associated receiver operating diagrams (29) are provided in Figure S2 in Supplementary Material.

To assess the test performance, the following formulae were used to calculate sensitivity and specificity (24), PPV and NPV (30), and PLR and NLR (31) from the result counts:

$$\text{Sensitivity}=\frac{\text{TP}}{\text{TP}+\text{TN}}$$

$$\text{Specificity}=\frac{\text{TN}}{\text{TN}+\text{FN}}$$

$$\text{PPV}=\frac{\text{TP}}{\text{TP}+\text{FP}}$$

$$\text{NPV}=\frac{\text{TN}}{\text{TN}+\text{FN}}$$

$$\text{PLR}=\frac{\text{Sensitivity}}{1-\text{Specificity}}$$

$$\text{NLR}=\frac{1-\text{Sensitivity}}{\text{Specificity}}$$

## Results

### Participants

The study was performed in 2015 with archived patient data that ranged from February 2012 until August 2015. We included 125 patients in this study with a mean age of 65 years (range 23–93 years). The study population included 47 female patients (38%). Twenty-four patients had an ACA territory stroke, 48 patients had a stroke in the superior division of the MCA territory, and 51 in the inferior division. The PCA territory stroke was diagnosed in 33 patients, and PICA territory infarctions occurred in 32 patients. The patient demographics are presented in Table 2.

**Table 2:** **Demographic characteristics of the study population (*n* = 125)**.

Characteristic	Value
Female sex – no. (%)	47 (38)
Age – years	
Mean ± SD	65 ± 15
Range	23–93
Diagnosed stroke territory – no. (%)	
ACA	24 (19)
MCA superior division	48 (38)
MCA inferior division	51 (41)
PCA	33 (27)
PICA	32 (26)

*ACA, anterior cerebral artery; MCA, middle cerebral artery; PCA, posterior cerebral artery; PICA, posterior inferior cerebellar artery*.

Of the total study cohort of 125 patients, 14 data sets were used to create the stroke territory reference map and were excluded from the test evaluation. Therefore, the analysis of our method’s diagnostic accuracy is based on 101 cases. These 101 cases were split into two groups of sizes 91 and 20. For cross-validation, the 91 cases were used and randomly divided into 10 subgroups on which 10 rounds of outer cross-validation were performed. In each round, the test parameters were optimized using the data of nine subgroups. Subsequently, the test performance was validated on the tenth subgroup each round. The performance results of all 10 rounds of cross-validation were then averaged. For an independent validation, the test parameters were optimized using the data of all 91 patients and then validated on the 20 independent data sets, which were excluded from cross-validation. Of those, 20 data sets were excluded from the cross-validation, to be later used for independent validation. The remaining 91 cases were randomly split up into 10 groups of 9 cases each (one group contained 10 cases). The study procedure is illustrated as a flow chart in Figure S3 in Supplementary Material.

The neurological diagnoses were approved by an experienced neurologist and neuroradiologist and were used as a reference standard. This reference and the stroke projection were both based on the same diffusion MRI dataset. Discrepancies in the test results were not ascribed to any change in the disease pattern.

Our study population had a median National Institute of Health Stroke Scale (NIHSS) Score (32–34) of 4 with an interquartile range of 9. As a measure of quantitative severity, we calculated the stroke per brain ratio from the diffusion MRI pixels and found that the study population had a mean of 4.3% (SD: 5.8%) of brain volume affected by stroke. The median of stroke per brain ratio was 2.3% with an interquartile range of 3.7%. For patients with strokes in the PCA territory, the quantitative severity even reached a median of 2.8% with an interquartile range of 5.4%. The most severely affected group of patients according to NIHSS was the one with infarctions in the superior division of the MCA territory. Here, the median score was 7 with an interquartile range of 13. A detailed list of the NIHSS Scores and quantitative severity for all brain artery territories is shown in Table 3.

**Table 3:** **Severity of stroke within the study population (*n* = 125)**.

	Quantitative severity (%)		NIHSS score	
	Median	Interquartile range	Median	Interquartile range
ACA	1.5	1.3	4	3
MCA superior division	1.9	1.9	7	13
MCA inferior division	1.6	2.1	2	6
PCA	2.8	5.4	3.5	5
PICA	2.0	5.2	1.5	2.5
Total	2.3	3.7	4	9

*The quantitative severity was calculated from the proportion of the number of diffusion MRI pixels representing stroke to the total number of “brain pixels”; NIHSS scores were taken from patient records*.

*ACA, anterior cerebral artery; MCA, middle cerebral artery; PCA, posterior cerebral artery; PICA, posterior inferior cerebellar artery*.

### Diagnostic Accuracy of Territory Recognition Based on Stroke Projections

The detection of stroke-affected brain artery territories based on geoprojected diffusion MRI data with our method reached a sensitivity of 81% (SD: 7.8%) and a specificity of 87% (SD: 6.8%) after cross-validation on 91 cases. In independent validation on another 20 cases, the test had a sensitivity of 75% with specificity of 86%. A detailed cross tabulation of the results for each round of cross-validation is given in Table S1 in Supplementary Material. The statistical analysis results are presented in Table 4.

**Table 4:** **Statistical analysis of stroke territory recognition based on the geoprojected, two-dimensional map compared to the clinical diagnoses**.

Outer cross-validation on 91 cases										
Validation group	Sensitivity	Specificity	PPV	NPV	PLR	NLR	TP	FP	TN	FN
1	84.6%	87.5%	0.733	0.933	6.769	0.176	11	4	28	2
2	75.0%	96.0%	0.938	0.828	18.750	0.260	15	1	24	5
3	86.7%	83.3%	0.722	0.926	5.200	0.160	13	5	25	2
4	76.9%	78.1%	0.588	0.893	3.516	0.295	10	7	25	3
5	64.3%	80.7%	0.600	0.833	3.321	0.443	9	6	25	5
6	78.6%	100.0%	1.000	0.912	0.000	0.214	11	0	31	3
7	92.3%	81.3%	0.667	0.963	4.923	0.095	12	6	26	1
8	83.3%	87.9%	0.714	0.935	6.875	0.190	10	4	29	2
9	75.0%	84.9%	0.643	0.903	4.950	0.295	9	5	28	3
10	88.9%	93.8%	0.889	0.938	14.222	0.119	16	2	30	2
Mean	80.6%	87.3%	0.749	0.906	6.853	0.225				
SD	7.8%	6.8%	0.137	0.042	5.258	0.097				

**Independent validation**										

On 20 cases	75.0%	85.5%	0.621	0.916	5.182	0.292	18	11	65	6

*Outer cross-validation was repeated 10 times with parameters resulting from the Youden Index optimization for nine of 10 randomly chosen subgroups and test performance validation on the tenth subgroups each. In independent validation, parameters were optimized on 91 cases and validated on 20 independent cases*.

*PPV, positive predictive value; NPV, negative predictive values; PLR, positive likelihood ratio; NLR, negative likelihood ratio; TP, true positive count; FP, false positive count; TN, true negative count; FN, false negative count; SD, standard deviation*.

In our study, we can rule out diagnostic review bias (35) because the diagnoses used as reference standards were already set before the study was conducted. Because the test software does not have any information about the reference standard results, there is also no test review bias (35).

The reference standard is affected by observer variability (36) because there is no standardized method for physicians to definitively diagnose the affected stroke area, whereas the projection method is fixed software that has no analytical noise.

## Discussion

Two-dimensional projections of three-dimensional data are a benefit because of their “at a glance” usability. Projection methods are commonly used to transform geographical data (spheres, planet surfaces) to maps that allow direct and two-dimensional analysis. In contrast to three-dimensional data, two-dimensional maps are immediately distinct without the need for dynamic presentation (e.g., continuous rotation of an object) or interaction (such as scrolling through stack image layers). These maps can be printed out in clinical reports without the loss of information on stroke position and size to provide a complete overview of the brain.

To assess projection quality, we compared the ratio of brain pixels covered by stroke in the original diffusion MRI data and in the two-dimensional projection. The projection of three-dimensional values onto a sphere is more accurate for near-surface values. Diffusion deficits that lie too deep in the brain cannot be projected properly and result in an oversized and scattered stroke across the two-dimensional map, which leads to a large difference in the stroke per brain ratios of the diffusion MRI and the projection as well as a loss of specificity. A possible way of addressing this problem would be to first project the inner MRI voxels onto a small sphere just around the center. Then in further steps, the voxels lying further outwards could be projected onto bigger spheres. Another weakness of the method is the step of mapping all projected MRI data points into the limited pixels of the two-dimensional maps. To ensure that the data lost during this process is information about healthy brain rather than the stroke position and size, each pixel of the map shows the maximum diffusion value of all data points that it represents. The histogram in Figure S4 in Supplementary Material shows, that this step is concerning about 40% of the map pixels. In many of them the maximum value is taken from a group of only a few data points. In some pixels though, the number of data points being represented by only one pixel on the map reaches up to about 35. Such losses are an inherent problem of data reduction methods and represent the cost of implementing a user-friendly and easily accessible two-dimensional map.

Since there still always is a loss of information and small infarctions could be missed, this method alone is not a reliable basis for clinical diagnosis. It can only add a way of illustrating the MR imaging results after careful inspection of the original imaging data by a physician. This step of visual inspection of the imaging data is inevitable to reach a reliable and safe diagnosis for the patients. The projection of strokes with our method will still serve a useful function as an easily accessible overview for reference for example in patient records and in research.

The projection may be weaker in representing inner and too small infarctions, yet it proves to be a useful tool for visualizing cortical damage and can be powerful in monitoring the progression of lesions over time. Moreover, it is compatible with other brain surface methods such as EEG. There is a plethora of different mathematical methods to project surface data to maps. Among these methods, the Mollweide projection is widely used in visible studies (37, 38). As an equal area projection, it has the advantage of representing projected areas in their correct size. The correct size is attained at the cost of distortions and users will need to adapt to the new topography. However, with the equal area method, the extent of cortical damage can be measured quantitatively.

When applying the technique, we found that the different brain artery territories are represented as intuitively comprehensible areas on the map. User friendliness is, of course, not enough to justify a new method. We therefore chose to apply the STARD criteria for reporting diagnostic accuracy as well as leave-p-out cross-validation and independent validation to substantiate the parameter thresholds. We find that with a sensitivity of 81% and a specificity of 87% in cross-validation and with sensitivity of 75% and specificity of 86% in independent validation, the detection of strokes in geoprojected brain maps can compete with diagnostic methods from other disciplines (39–42). The specificity could be improved in the future by using a more accurate and complete stroke territory reference map based on a larger number of patients. These small error rates in the brain artery detection show that strokes are presented sufficiently correct in the geoprojected two-dimensional maps for them to be a useful visualization tool.

Diffusion MR imaging is the gold standard for illustrating stroke lesions, especially in the acute phase. We have reported a new method for presenting these imaging data. There is no additional data acquisition needed, and on a standard current office computer, the projection of one dataset takes approximately 3 min. Our method only adds to the visualization possibilities of diffusion MRI data without imposing any additional risks or inconveniences on patients or physicians.

In summary, our analytical method complements the classical approach in terms of intuitiveness, time and repeatability compared to pure subjective visual inspection. Whereas the possibility of presenting and printing a comprehensive overview of the brain all at once is a big advantage, one can also imagine this method could be useful in the future for studies comparing and overlaying diffusion MRI results with other brain imaging methods as well as surface methods such as EEG. Altogether, this work contributes to furthering the applicability of diffusion MRI data in ischemic stroke territory recognition.

## Author Contributions

First and corresponding author JKW carried out the main part of the work including programing the processing scripts, all statistical analyses and wrote the manuscript. BV supported this work by approving clinical diagnoses taken from patient records. He contributed to the study design and critically revised the manuscript. PG and DFG kindly revised the manuscript and provided helpful comments to improve the study design. AD and SS provided the necessary MRI data and gave helpful feedback on the manuscript. JK critically revised the manuscript and provided helpful comments; also he provided the lab’s research environment in which this work developed. TWG provided the idea for this work. He supervised the working process and revised the manuscript.

## Conflict of Interest Statement

The authors declare that the research was conducted in the absence of any commercial or financial relationships that could be construed as a potential conflict of interest.
