# Corrigendum: Feasibility and Diagnostic Accuracy of Ischemic Stroke Territory Recognition Based on Two-dimensional Projections of Three-dimensional Diffusion MRI Data

**DOI:** 10.3389/fneur.2017.00056

**Published:** 2017-02-20

**Authors:** Jana Katharina Wrosch, Bastian Volbers, Philipp Gölitz, Daniel Frederic Gilbert, Stefan Schwab, Arnd Dörfler, Johannes Kornhuber, Teja Wolfgang Groemer

**Affiliations:** ^1^Department of Psychiatry and Psychotherapy, Friedrich-Alexander University of Erlangen-Nuremberg, Erlangen, Germany; ^2^Department of Neurology, Friedrich-Alexander University of Erlangen-Nuremberg, Erlangen, Germany; ^3^Department of Neuroradiology, Friedrich-Alexander University of Erlangen-Nuremberg, Erlangen, Germany; ^4^Institute of Medical Biotechnology, Friedrich-Alexander University of Erlangen-Nuremberg, Erlangen, Germany; ^5^Psychiatric and Neurological Ambulatory Care Office, Bamberg, Germany

**Keywords:** computer-aided detection and diagnosis, diffusion-weighted imaging, dimensionality reduction, magnetic resonance imaging, stroke territories, validation, visualization

In the original article, we neglected to state that this work was performed in partial fulfillment of the requirements for obtaining the degree “Dr. rer. biol. hum.” at the University of Erlangen-Nuremberg. The authors apologize for this oversight.

This error does not change the scientific conclusions of the article in any way.

## Conflict of Interest Statement

The authors declare that the research was conducted in the absence of any commercial or financial relationships that could be construed as a potential conflict of interest.

