# Supplementary material for: Feasibility and Diagnostic Accuracy of Ischemic Stroke Territory Recognition Based on Two-Dimensional Projections of Three-Dimensional Diffusion MRI Data
Source: Front Neurol. 2015 Nov 19;6:239. doi: 10.3389/fneur.2015.00239 (PMC4652171; doi:10.3389/fneur.2015.00239)
Supplement: Supplementary file 1 [file data_sheet_1.pdf]

Supplementary Material

Feasibility and diagnostic accuracy of ischemic stroke territory recognition based on two-dimensional projections of three-dimensional diffusion MRI data

Wrosch, J.K.*, Volbers, B., Gölitz, P., Gilbert, D.F., Schwab S., Dörfler, A., Kornhuber, J., Groemer, T.W.

*** Correspondence:** Jana Katharina Wrosch: jana.wrosch@uk-erlangen.de

1. Supplementary Figures and Tables

1.1. Supplementary Figures

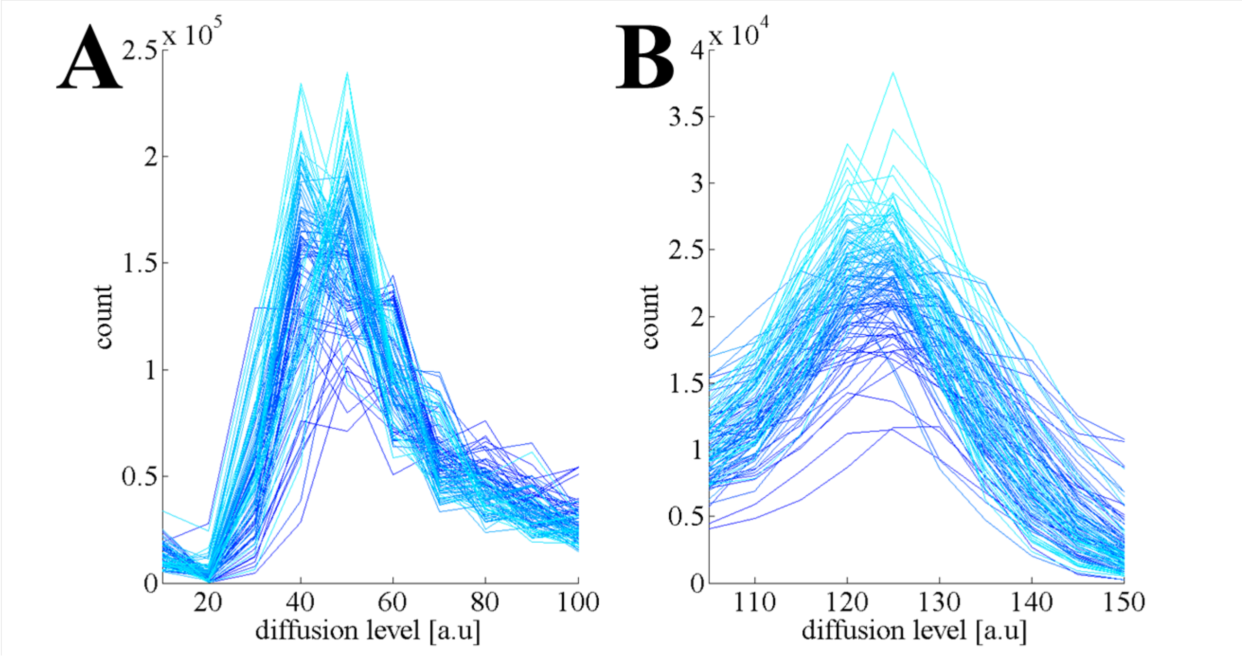

Supplementary Figure 1. Enlarged view of the value distributions in the diffusion level histogram: A) Distribution of the diffusion levels representing the background; B) Distribution of the diffusion levels representing the brain.

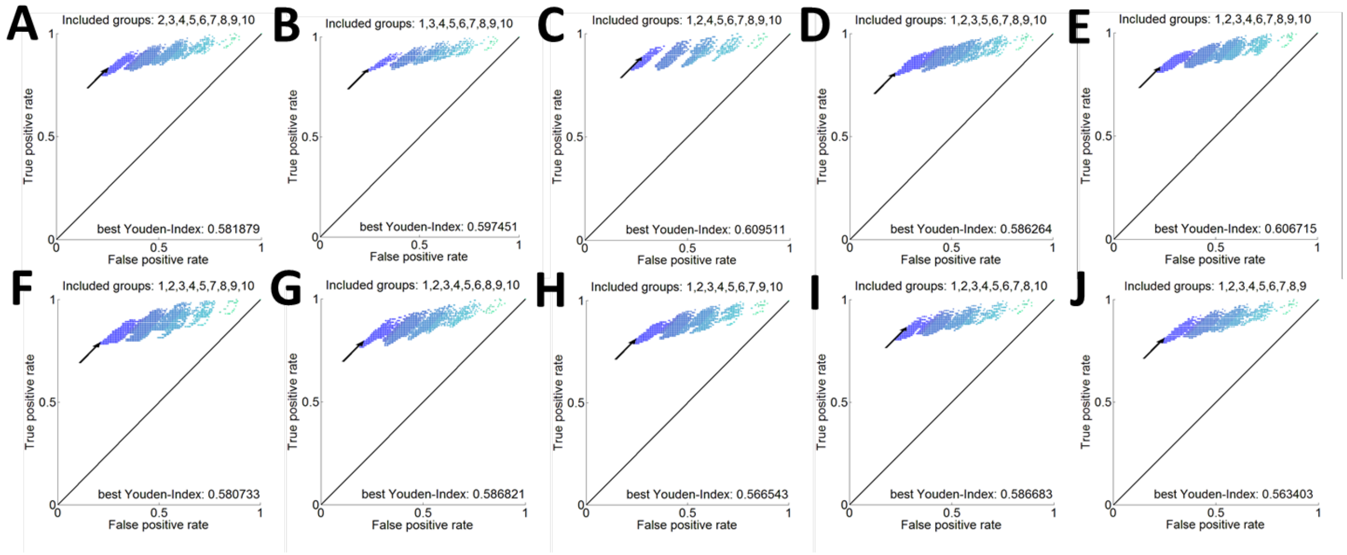

Supplementary Figure 2. Optimization of cut-off values for computer-aided brain artery territory diagnosis: A-J) Receiver operating diagrams of all possible combinations of cut-off values of the ten test parameters (two criteria for five territories each) for each of the ten outer cross-validation rounds. The arrow marks the point with maximum Youden-Index in each diagram. The parameters corresponding to this point were used as optimized cut-off values for the brain artery territory recognition and are listed in Table 1. The optimization is based on all ten randomly filled subgroups except: A) group 1; B) group 2; C) group3; D) group 4; E) group 5; F) group 6; G) group 7; H) group 8; I) group 9; J) group 10.

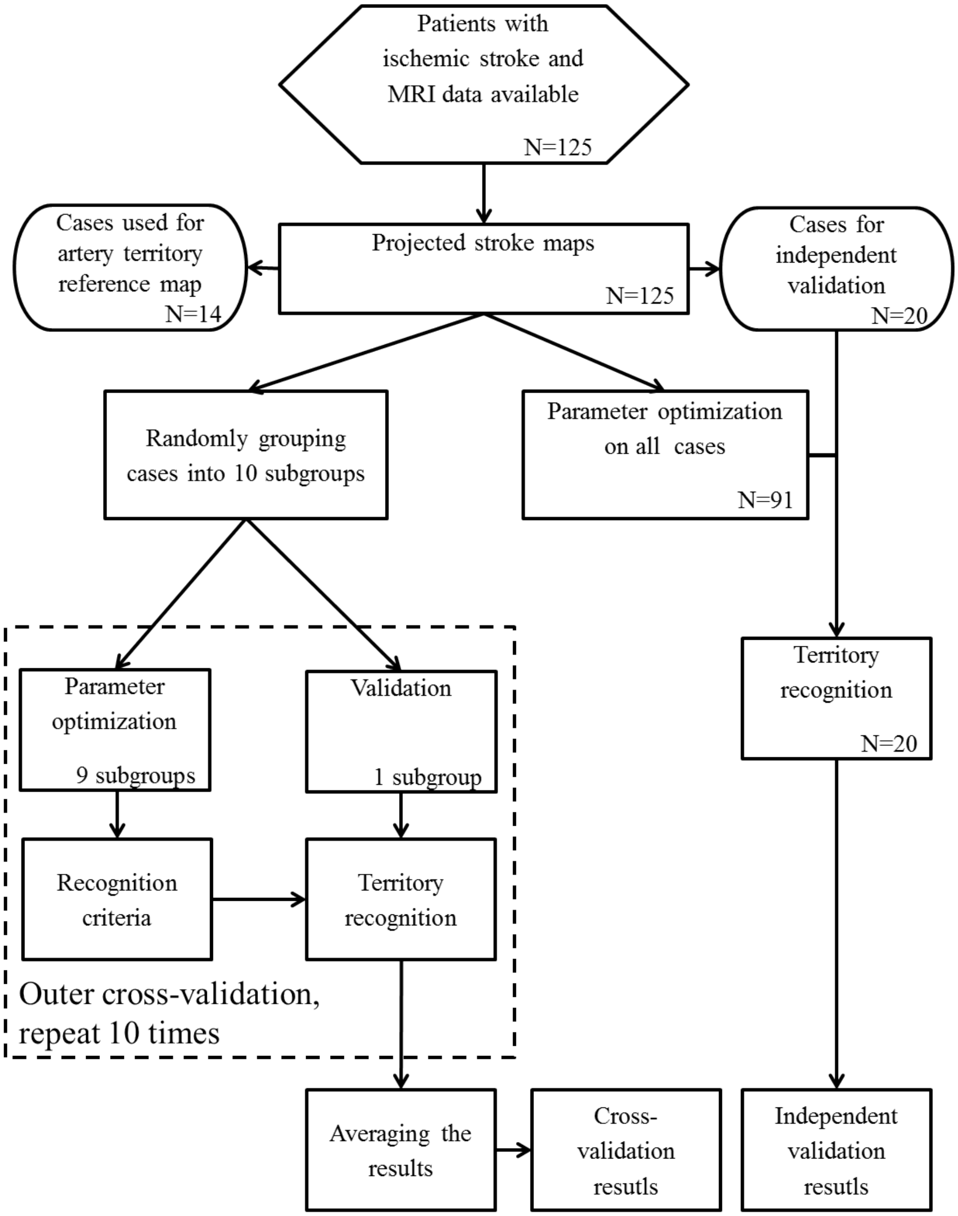

Supplementary Figure 3. Flow diagram of the study procedure. 125 patients were included in the study. All these patients' diffusion MRI data sets were projection to two-dimensional maps. Of these 125 maps, 14 were used to create the brain artery territory reference and were excluded from further analysis. The remaining 101 cases were split into two groups of sizes 91 and 20. For cross validation the 91 cases were used and randomly divided into ten subgroups on which 10 rounds of outer cross-validation were performed. In each round the test parameters were optimized using the data of 9 subgroups. Subsequently, the test performance was validated on the tenth subgroup each round. The performance results of all ten rounds of cross-validation were then averaged. For an independent validation, the test parameters were optimized using the data of all 91 patients and then validated on the 20 independent data sets, which were excluded from cross-validation.

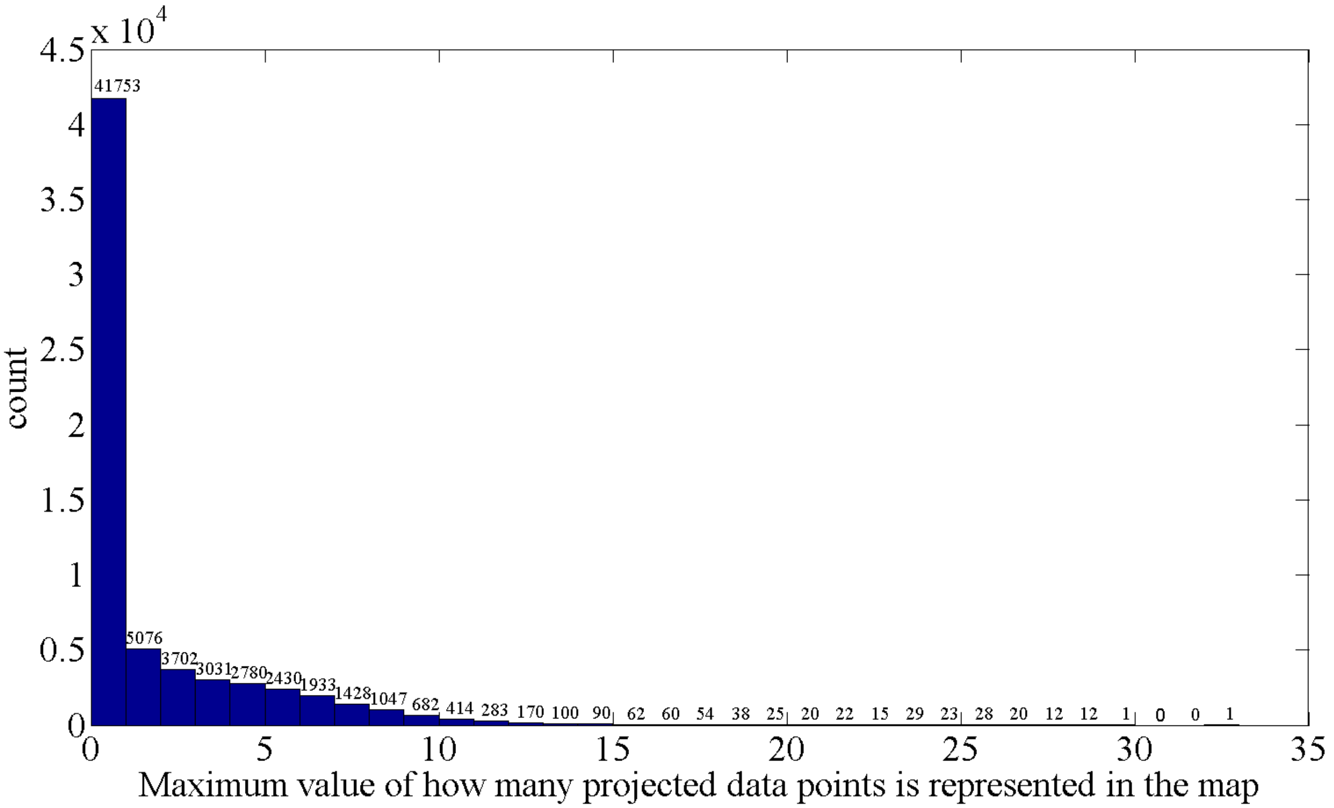

Supplementary Figure 4. Mapping all MRI data points onto the limited pixels in the two-dimensional map. Histogram showing, how many MRI data points are integrated into each pixel of the two-dimensional map. The value on the map is representing the maximum of all integrated data points each.

1.2. Supplementary Tables

Supplementary Table 1. Cross tabulation of the the positive and negative test result counts for each of ten outer cross-validation steps. The territory recognition is based on the geoprojected, two-dimensional map and the clinical diagnoses used as reference standards. Recognition parameters resulted from Youden Index and ROC area under the curve optimization on 9 randomly filled subgroups. Result counts show the test performance on the tenth subgroup each. ACA: Anterior cerebral artery territory; supMCA: superior division of middle cerebral artery territory; infMCA: inferior division of middle cerebral artery territory; PCA: posterior cerebral artery territory; PICA: posterior inferior cerebellar artery territory.

Diagnosed infarction	Territory recognition test results																			
	Group 1		Group 2		Group 3		Group 4		Group 5		Group 6		Group 7		Group 8		Group 9		Group 10	
	+	-	+	-	+	-	+	-	+	-	+	-	+	-	+	-	+	-	+	-
ACA	2	0	3	2	2	0	1	2	2	0	0	0	2	0	1	0	1	0	0	2
no ACA	0	7	0	4	2	5	1	5	2	5	0	9	1	6	2	5	0	8	2	6
supMCA	2	0	4	0	5	1	2	0	4	0	2	1	3	0	3	0	4	0	5	0
no supMCA	3	4	0	5	0	3	1	6	1	4	0	6	1	5	0	6	1	4	0	5
infMCA	4	0	3	1	2	1	2	0	1	3	4	1	4	0	2	1	1	1	6	0
no infMCA	0	5	1	4	0	6	1	6	0	4	0	4	1	4	0	6	1	6	0	4
PCA	0	1	3	0	2	0	2	1	1	1	3	1	2	0	2	0	1	1	3	0
no PCA	1	7	0	6	3	4	2	4	2	5	0	5	1	6	1	6	2	5	0	7
PICA	3	1	2	2	2	0	3	0	1	1	2	0	1	1	1	1	2	1	2	0
no PICA	0	5	0	5	0	7	2	4	1	6	0	7	2	5	1	6	1	5	0	8
